# Artificial Intelligence Replacement Dysfunction (AIRD): A Call to Action for Mental Health Professionals in an Era of Workforce Displacement

**DOI:** 10.7759/cureus.93026

**Published:** 2025-09-23

**Authors:** Stephanie N McNamara, Joseph E Thornton

**Affiliations:** 1 Department of Psychiatry, University of Florida College of Medicine, Gainesville, USA

**Keywords:** aird, ai replacement, artificial intelligence, job displacement, mental health, occupational identity, psychiatry

## Abstract

Artificial intelligence replacement dysfunction (AIRD) is a new, proposed clinical construct describing the psychological and existential distress that could be experienced by individuals facing the threat or reality of job displacement due to artificial intelligence (AI). As AI systems increasingly automate tasks across industries, workers may present to mental health professionals with symptoms such as anxiety, insomnia, depression, or identity confusion symptoms that may reflect deeper fears about relevance, purpose, and future employability. This paper introduces AIRD as a conceptual framework for understanding these presentations, outlines common symptom patterns, proposes practical tools for screening and intervention, and suggests an imperative for advocacy. We describe therapeutic strategies, including motivational interviewing, narrative therapy, occupational identity restructuring, and adaptation. Additionally, we urge clinicians to take on systems-level advocacy roles in shaping institutional, educational, and policy responses to AI-related workforce disruptions. For clinical practitioners, increasing awareness of AIRD may improve therapeutic recognition and intervention. As AI transforms the labor landscape, mental health professionals must be prepared to recognize and respond to the emotional and social consequences it leaves in its wake.

## Introduction

The rapid developments in artificial intelligence (AI) have revolutionized tasks across a wide array of industries, impacting both blue- and white-collar professions. From generative AI systems drafting legal documents to autonomous technologies replacing roles in manufacturing and logistics, the human labor landscape is shifting at a profound scale. According to industry leaders such as Dario Amodei, CEO of Anthropic, AI may replace up to 50% of entry-level white-collar jobs within five years [1]. Other sources claim these projections are wildly overestimated [2]. For example, the Future of Jobs report projects that the loss of 92 million jobs will be offset by a potential gain of 170 million jobs [3]. However, at least one strong study from Stanford reports that there will be significant, although unequal, job loss impacting large segments of our society [4]. This unprecedented change threatens not just economic stability but also individual identity and psychological well-being. Mental health professionals must prepare for a growing cohort of patients experiencing distress not rooted in traditional psychopathology, but in the existential threat of professional obsolescence [5]. We define this presentation as artificial intelligence replacement dysfunction (AIRD) and propose a clinical framework to guide its recognition and treatment.

## Technical report

Clinical model for AIRD

AIRD may manifest through a range of psychological symptoms, including anxiety, insomnia, demoralization, and a profound loss of occupational identity [6]. Clinically, AIRD is characterized by an intense and persistent fear of job loss or personal obsolescence resulting from AI integration, often leading to significant functional impairment. Affected individuals may experience significant cognitive and emotional shifts, such as paranoia and feelings of worthlessness, resentment, and hopelessness. These symptoms typically arise in the absence of other primary psychiatric disorders, substance use, or structural brain pathology. They are closely tied to situational stressors, such as layoffs, role displacement, or major technological shifts in the workplace.

Variations in AIRD presentation

As we begin to observe the psychological impact of AI-related job displacement, we predict that individuals will not experience AIRD in a uniform way. Rather than offering formal diagnostic subtypes, we describe a range of symptom constellations, each reflecting a distinct way people may react to the perceived or real threat of professional obsolescence. These patterns can help clinicians recognize emerging themes in distress and tailor their interventions accordingly. Many of the symptoms may revolve around feelings of irrelevance in the workplace, including professional identity loss, inadequacy, and loss of purpose. Other symptoms may be low self-worth, an obsession with efficiency, and denial of AI's relevance as a defense mechanism.

People with these patterns may present initially with complaints like insomnia and stress that, on investigation, are due to AIRD. One study showed that sleep disorders like insomnia are associated with workers' feelings of job insecurity due to technological innovation [7]. Another recent study found that negative emotional responses like stress are common for workers who are susceptible to professional obsolescence due to AI [8]. For mental health professionals, recognizing these symptoms and patterns can guide therapeutic conversations, inform supportive interventions, and help clinicians validate the deep psychological impact of a rapidly changing world.

Screening

Screening for AIRD requires a nuanced, contextual approach that recognizes the overlap between common symptoms of anxiety or depression and the emerging psychological responses to AI-driven job displacement. A 2025 study found a positive correlation between AI implementation in the workplace and anxiety and depression [7]. While AIRD is not yet a formal DSM diagnosis, clinicians can screen for it by incorporating specific questions into standard assessments, focusing on work-related distress, existential themes, and AI-related fears.

We propose a structured approach for screening. Begin with a contextual inquiry through open-ended questions that explore the patient's current work situation and their perception of AI and automation, such as the following: "Can you tell me about any recent changes in your job or industry?", "How do you feel about the increasing use of artificial intelligence in your field?", and "Have you ever felt that your job might be at risk due to automation or AI?".

These types of questions help to uncover latent distress without immediately pathologizing it. Next, identify core emotional and cognitive themes for reactions linked to AIRD. Look for signs and symptoms of anxiety such as fear of job loss or future uncertainty. Also, look for demoralization associated with loss of purpose or feelings of redundancy. Then explore for disruption of identity, especially confusion about self-worth apart from occupational identity. Finally, consider that due to displacement, grief may mask the mourning of a role or career trajectory. Sample probing questions could be as follows: "How would you describe your sense of purpose in your work right now?" or "Do you feel your skills or experience are still valued?".

While no validated AIRD-specific tool exists yet, clinicians could adapt elements from existing measures. Examples could include occupational subscales from the Patient Health Questionnaire-9 (PHQ-9) (e.g., "Feeling down, depressed, or hopeless about your work"), work stress items from the Generalized Anxiety Disorder-7 (GAD-7), and post-traumatic stress disorder (PTSD) analogues (e.g., intrusive thoughts about career loss, avoidance of industry news, hypervigilance about job metrics) [9,10].

The authors offer a proposed AIRD screening questionnaire in Table 1.

**Table 1:** AIRD screening questionnaire Future studies are needed to validate the screening tool and to define a cut-off score. An AIRD score of ≥10 may indicate AIRD-related distress warranting further exploration. AIRD: artificial intelligence replacement dysfunction

Item	0	1	2	3	4	Total
I worry that my job or profession will be replaced by artificial intelligence.						
I feel that my skills are becoming obsolete due to new technologies.						
I find myself avoiding news or conversations about automation or artificial intelligence.						
I have trouble imagining a meaningful future in my career.						
I feel emotionally distressed when thinking about changes in my workplace due to artificial intelligence.						
Total score						

When evaluating individuals for AIRD, it is essential to rule out other potential causes of their symptoms. Clinicians should assess for pre-existing mood or anxiety disorders, cognitive impairment, situational stressors unrelated to AI, and substance use, especially when AIRD symptoms resemble those of major depressive disorder or adjustment disorder. Even if AIRD does not meet the full diagnostic criteria for a formal mental health condition, the emotional experience is genuine and deserves therapeutic attention. These kinds of concerns are becoming more common as AI reshapes how many people think about their careers and futures. 

## Discussion

Treatment considerations

The management of AIRD requires an integrative, human-centered approach that acknowledges both the real-world changes occurring in the labor market and the deeply personal, often existential, impact these changes have on individual identity. Clinicians should be prepared to address AIRD not as a pathology in isolation, but as a psychological response to social and technological upheaval.

The first task is to create a therapeutic space where patients feel heard and understood. Acknowledging that fears about job loss, obsolescence, or identity collapse are legitimate responses to real changes can reduce shame and alienation. However, clinicians must walk a careful line to validate distress while also encouraging adaptive responses rather than passivity or despair.

Patients experiencing AIRD may feel stuck or overwhelmed. Motivational interviewing techniques can help them explore ambivalence, identify intrinsic values, and begin to imagine possible paths forward [11]. By framing change as a personal choice rather than an external imposition, clinicians can help patients shift from helplessness to agency.

Many individuals derive a significant portion of their self-worth from professional identity. A recent study suggests that factors like an increase in unemployment due to AI-driven job displacement will have adverse effects on the mental health of the population at large because of economic instability [12]. Therapy should support patients in expanding their sense of self beyond job titles or economic productivity. This may involve revisiting earlier interests, exploring relational roles (e.g., mentor, parent, volunteer), or cultivating aspects of identity that AI does not replicate, such as empathy, creativity, or moral insight.

Narrative Therapy and Cognitive Restructuring Techniques

Patients struggling with AIRD are likely to carry stories of irrelevance or inadequacy. Narrative therapy helps externalize those beliefs and reframe them in a more empowering light. Cognitive restructuring can challenge automatic negative thoughts, such as "I am useless now" or "Everything I built is obsolete", and replace them with more accurate and compassionate interpretations. These techniques help patients build psychological resilience and restore a coherent sense of self [5,13].

Short-Term Behavioral Strategies for Insomnia or Anxiety

The preferred interventions for insomnia or anxiety are Cognitive Behavioral Therapy for Insomnia (CBT-I), mindfulness-based stress reduction, relaxation training, and structured routines that can address common somatic symptoms associated with AIRD [14,15]. These tools also empower patients to take active steps toward self-regulation and emotional grounding.

Advocacy

Clinicians have a powerful opportunity, and arguably a responsibility, to play systems-level advocacy roles in addressing AIRD [16]. Beyond the clinical office, clinicians can help shape institutional policies, public discourse, and workforce strategies that acknowledge and mitigate the mental health impact of AI-driven job displacement. Here are key systems advocacy roles clinicians can assume:

Institutional Advocate for Workforce Mental Health 

Promoting awareness of AIRD within healthcare systems, universities, and professional organizations will be a key feature, as will encouraging Human Resources (HR) departments and Employee Assistance Programs (EAPs) to incorporate AIRD awareness into their workforce wellness plans. Additionally, training sessions can be effective for both clinical and administrative staff in recognizing AI-related distress. Internal screening protocols and referral pathways can also be developed or promoted for workers struggling with AI-induced vocational anxiety [17].

Educator in Professional Training Programs and Researcher

The clinician advocates are tasked with integrating AIRD into medical, psychology, social work, and counseling education, while also developing case-based curricula or simulation exercises focused on AI-related occupational distress. A critical feature will include training future mental health professionals to identify, assess, and treat AIRD in diverse populations [18]. Researchers can further contribute to the evidence base and conceptual development of AIRD, conduct qualitative or mixed-methods research on the psychological effects of AI displacement, and publish case reports, position papers, or pilot studies that validate AIRD screening tools or interventions.

Consultants to Employers and Unions 

The clinician advocates are entrusted with helping companies and labor organizations support psychologically healthy adaptation to AI to ensure worker well-being. This person can serve as a consultant on workforce transition planning (e.g., emotional support during layoffs, post-redeployment counseling). Finally, partnering with unions to ensure that the psychological risk of AIRD and automation is addressed in collective bargaining discussions will provide employee protection [16].

Policy Advocate

A key advocacy task will be to inform and influence policy decisions related to labor, education, and mental health infrastructure. Other roles may involve submitting expert testimony or consulting on legislative efforts related to AI regulation, workforce retraining, or mental health parity and advocating for inclusion of mental health services in state and federal economic transition programs [19]. A principal aspect can include conducting public outreach using social media and other media appearances to raise awareness of AIRD as a legitimate mental health concern.

## Conclusions

AIRD represents a critical and emerging challenge for the field of mental health that warrants further research. As AI technologies rapidly alter the nature of work across sectors, clinicians will increasingly encounter patients experiencing distress not due to a primary psychiatric disorder, but as a reaction to the threat or reality of job displacement, identity erosion, and existential uncertainty. These individuals may present with symptoms of anxiety, depression, insomnia, or demoralization, yet their suffering is deeply rooted in the destabilizing effects of AI-driven labor change. Equipping mental health professionals with the knowledge and tools to recognize and treat people with AIRD will be vital for societal acceptance of a condition that will increasingly affect the workplace. As the boundaries between human work and machine capability continue to blur, we must ensure that our clinical, educational, and societal systems are prepared not just to keep pace with innovation but to care for the people it displaces. Recognizing and responding to AIRD is a step toward that future.
